# The effect of bariatric surgery on metabolic syndrome: A retrospective cohort study in Colombia

**DOI:** 10.1002/hsr2.1090

**Published:** 2023-01-30

**Authors:** Gonzalo A. Domínguez Alvarado, María C. Otero Rosales, Julián C. Cala Duran, Sergio Serrano‐Gómez, Tania Y. Carrero Barragan, Paula N. Domínguez Alvarado, Martha L. Ramírez, Kamila Serrano‐Mesa, Ivan D. Lozada‐Martinez, Alexis R. Narvaez‐Rojas, Luis E. López Gómez

**Affiliations:** ^1^ Grupo de Innovación e Investigación Quirúrgica, School of Medicine Universidad Autónoma de Bucaramanga Bucaramanga Colombia; ^2^ School of Medicine Universidad Industrial de Santander Bucaramanga Colombia; ^3^ School of Medicine Universidad de Santander Bucaramanga Colombia; ^4^ Department of Bariatric Surgery Clínica FOSCAL Internacional Floridablanca Colombia; ^5^ Medical and Surgical Research Center, Future Surgeons Chapter, Colombian Surgery Association Bogotá Colombia; ^6^ Grupo Prometheus y Biomedicina Aplicada a las Ciencias Clínicas, School of Medicine Universidad de Cartagena Cartagena Colombia; ^7^ International Coalition on Surgical Research Universidad Nacional Autónoma de Nicaragua Managua Nicaragua; ^8^ Breast Surgical Oncology Division, DeWitt Daughtry Family Department of Surgery Jackson Health System/University of Miami Miller School of Medicine Miami Florida USA

**Keywords:** bariatric surgery, cardiometabolic risk factors, colombia, metabolic syndrome, obesity

## Abstract

**Introduction and Objective:**

Metabolic syndrome (MetS) represents a group of metabolic abnormalities. It is currently a pandemic, and its prevalence is on the rise. MetS has a direct relationship with obesity, for this reason, bariatric and metabolic surgery has been proposed as a method to simultaneously control obesity and MetS. However, in Colombia the results of this intervention are unknown. This study aims to compare metabolic syndrome before and after bariatric surgery in a Colombian population.

**Methods:**

Retrospective cohort study conducted in a highly complex institution in Colombia, where comparing the prevalence of metabolic syndrome in patients who underwent bariatric surgery during a 1‐year follow‐up period, between January 2015 and December 2019. Of these patients, 48 underwent Roux‐en‐Y gastric bypass, and 32 underwent sleeve gastrectomy by laparoscopic technique.

**Results:**

A total of 80 patients were included, of which 67.5% were women and the mean age was 42.8 ± 12.9 years. The most frequent preprocedure comorbidities were arterial hypertension (36.2%), dyslipidemia (32.4%), and sleep apnea (20%). After bariatric surgery, the prevalence of metabolic syndrome decreased from 66.2% to 3.7% (*p* < 0.05). In addition, a reduction in the Homeostatic Model Assessment for Insulin Resistance score from 77.5% to 22.5% was observed during the follow‐up period. HbA1c, creatinine, and thyroid‐stimulating hormone, were the only parameters without significant changes.

**Conclusions:**

Metabolic and bariatric surgery is an effective treatment for weight reduction, with a high impact in reducing the prevalence of metabolic syndrome and insulin resistance in the short and medium term in the Colombian population.

## 1 INTRODUCTION

Metabolic Syndrome (MetS) represents a group of metabolic abnormalities including arterial hypertension, central obesity, insulin resistance (IR), atherogenic dyslipidemia among others. It is strongly associated with obesity; however, it is not always synonymous with MetS.^1^ The MetS is currently a pandemic; its prevalence varies worldwide and often corresponds to the prevalence of obesity. MetS affects an estimated 150 million people in Europe and 65 million in the United States, Southeast Asia has a prevalence of 22.9%.^2^ In Latin America, MetS has a prevalence of 15% in people over 20 years of age.^3^ In Colombia, it has been estimated that the urban prevalence of MetS among men and women is 9% and 19%, respectively.^4^ Pinzón et al.,^5^ in 2007 estimated that the prevalence of MetS in an adult population of the city of Bucaramanga was 12.3% according to the Adult Treatment Panel III (ATP III) classification and 32.9% with the classification of the IDF. Since then, this phenomenon has not been studied again.^2^, ^6^

The MetS has a strong relationship with obesity, a pathology with high prevalence in the world and difficult to manage.^7^ In the last decade, increasing economic resources have been directed to develop new strategies that can control the rise in prevalence of obesity, however, the established goals have not been met.^8^ There are multiple treatments proposed for the management of MetS, from the increase in physical activity, to pharmacological management, even so, in many cases a weight loss of more than 10% is not achieved, and those who manage to lose weight, on multiple occasions recover the weight lost quickly.^9^

Currently, Metabolic and Bariatric Surgery (MBS) is the indicated treatment for obesity when other measures have failed, since it is a safe and effective procedure, additionally it is supported by multiple academic consensuses and the National Institute of Health of the United States.^10^ However, there is a paucity of literature on the effect of bariatric surgery on the MetS. In Colombia, the results of this intervention are unknown. This study aims to compare metabolic syndrome (specific objectives of clinical parameters) before and after bariatric surgery in a Colombian population.

## 2 PATIENTS AND METHODS

### 2.1 Study design and patient selection

The reporting of this study conforms to the STROBE guidelines.^11^

Retrospective cohort study of anonymized secondary data, whose follow‐up begins from the indication of the surgical procedure to 1 year postoperatively. A population of patients undergoing MBS was taken from a highly complex institution specialized in bariatric surgery in the city of Bucaramanga and its metropolitan area, Colombia, between January 2015 and December 2019.

Inclusion criteria: Patients between 18 and 80 years old, BMI > 30 kg/m^2^, strict follow‐up at 1 year of the clinical variables already exposed. Exclusion criteria: Patient with previous bariatric surgeries. In addition, the type of procedure to be performed was taken from the management guidelines of the Colombian Association of Obesity and Bariatric Surgery.

### 2.2 Data extraction and variable definition

Demographic variables (weight, body mass index [BMI], waist hip index, type 2 diabetes mellitus, arterial hypertension (hypertension), dyslipidemia, sleep apnea, coronary heart disease, deep vein thrombosis, gastroesophageal reflux, depression, dermatitis, cholelithiasis) and clinical variables (glycosylated hemoglobin [HbA1c], basal glycemia, triglycerides, total cholesterol, high‐density lipoproteins (HDL), low‐density lipoproteins, creatinine, uric acid, thyroid‐stimulating hormone (TSH) and insulinemia) which were evaluated before and 12 months after the procedure.

According to the variables described, the diagnosis of MetS was defined by an internist following the criteria established in the International Diabetes Federation (IDF) scale^12^; Increase in waist circumference with specificity with respect to different ethnic groups; (man > 90 cm and woman > 80 cm), increased triglycerides: ≥1.7 mmol/L (150 mg/dL) or specific treatment of this lipid alteration, decrease in HDL cholesterol <1.03 mmol/L (40 mg/dL) in men <1.29 mmol/L (50 mg/dL) in women or specific treatment of this lipid alteration, increased systolic (≥130 mmHg) or diastolic (≥85 mmHg) blood pressure or treatment of previously diagnosed hypertension. A MetS diagnosis required ≥3 criteria.

### 2.3 Statistical analysis

A descriptive analysis was performed using the mean and standard deviation for continuous variables (all continuous variables had a normal distribution evaluated by the Shapiro–Wilk test), categorical variables were described by absolute frequencies and relative frequencies. A bivariate analysis was performed comparing the pre‐surgical and postsurgical values (per year), the continuous variables were compared using a paired mean difference test, the categorical variables were compared by a test of differences of paired proportions. A *p* < 0.05 was defined as statistical significance. All analyses were performed using stata 15.0.

### 2.4 Ethical statements

It is an observational project based on anonymized secondary data. This study was reviewed and approved by the Ethics Committee of the Universidad Autónoma de Bucaramanga (no. INV2‐FO‐01).

## 3 RESULTS

During the period from January 2015 to December 2019, 209 MBS were carried out. After applying the inclusion and exclusion criteria, 80 patients were included. Of the total participants, 54 (67.5%) were women and the mean age of the population was 42.8 ± 12.9 years. Two types of techniques were performed: Laparoscopic Roux–in–Y Gastric Bypass (LRYGB) in 48 participants and Sleeve Gastrectomy (SGIT) by laparoscopic technique in 32 participants. The most frequent pre‐procedure comorbidities were: arterial hypertension (36.2%), dyslipidemia (32.4%), sleep apnea (20%), and diabetes mellitus (12.4%) (Table 1).

**Table 1:** Distribution of sociodemographic and clinical variables of the study population.

	SGIT	LRYGB	Total
	*n* (%)	*n* (%)	*n* (%)
Number of patients	32 (40)	48 (60)	80 (100)
Gender			
Female	20 (62.5)	34 (70.8)	54 (67.5)
Male	12 (37.5)	14 (29.1)	26 (32.5)
Age (years)^a^	39.93 ± 12	44.8 ± 13.2	42.8 ± 12.9
Comorbidities			
Arterial hypertension	8 (25)	21 (43.7)	29 (36.2)
Dyslipidemia	13 (40.6)	13 (27)	26 (32.4)
Sleep apnea	5 (15.6)	11 (22.9)	16 (20)
Type 2 diabetes mellitus	1 (3.1)	9 (18.7)	10 (12.4)
Cholelithiasis	4 (12.5)	5 (10.4)	9 (11.2)
Depression	2 (6.2)	6 (12.5)	8 (10)
Coronary heart disease	‐	3 (6.2)	3 (3.7)
Gastroesophageal reflux	‐	3 (6.2)	3 (3.7)
DVT	‐	2 (4.1)	2 (2.5)
Dermatitis	2 (6.2)	‐	2 (2.5)
Metabolic syndrome	20 (62.5)	33 (68.7)	53 (66.2)

Abbreviations: DVT, deep vein thrombosis; LRYGB, Laparoscopic Roux–in–Y Gastric Bypass; SGIT, sleeve gastrectomy.

^a^ Mean ± standard deviation.

All variables were evaluated pre‐procedure and after 1 year of the surgical procedure, the vast majority had a statistically significant decrease, except HbA1c, creatinine, and TSH (Table 2). HbA1c was not statistically significant since there were values in extreme ranges that could affect this significance. However, a wide change in the mean HbA1c pre‐ and postprocedure can be noted. None of the patients who participated in the study had a history of kidney disease or altered preprocedure creatinine values, which is why there were no significant changes after the procedure, it should be emphasized that there was no alteration of the same, which reflects that the procedure is not a risk factor for kidney failure.

**Table 2:** Comparative results of postoperative follow‐up at 12 months.

Parameter	Pre‐surgical	Follow‐up at 12 months	*p* Value
	Mean ± SD (95% CI)	Mean ± SD (95% CI)	
*Vital signs*			
Heart rate, mm Hg	88.7 ± 12.8 (12.7–85.9)	77.1 ± 10.8 (10.8–74.7)	<0.001
Systolic blood pressure, mm Hg	127.2 ± 13.7 (124.3–130.4)	114.3 ± 12.2 (111.5–117)	<0.001
Diastolic blood pressure, mm Hg	80.8 ± 8.9 (78.7–82.6)	71.8 ± 6.8 (70.3–73.4)	<0.001
Mean blood pressure, mm Hg	97.4 ± 14.6 (94.2–100.7)	83.5 ± 15.4 (80.1–87)	<0.001
*Anthropometric measurements*			
BMI, kg/m^2^	39.4 ± 5.4 (38.2–40.6)	27.1 ± 3.8 (26.2–27.9)	<0.001
Waist, cm	122.5 ± 14.4 (119.8–126.5)	93.1 ± 11 (90.6–95.6)	<0.001
Hip, cm	130.5 ± 11 (127.3–132.5)	105.6 ± 8.8 (103.4–107.4)	<0.001
Waist/hip index, cm	0.9 ± 0.1 (0.9–0.9)	0.8 ± 0.1 (0.8–0.9)	<0.001
% Fat	46.1 ± 7.8 (44.3–47.8)	30.9 ± 9.8 (28.5–32.9)	<0.001
*Laboratory tests*			
HbA1c	6.3 ± 4.5 (5.2–7.3)	5.2 ± 0.5 (5.1–5.3)	0.053
Basal glycemia	97.2 ± 17.2 (93.3–101)	83.8 ± 9.6 (81.7–86)	<0.001
Triglycerides	157.1 ± 64.1 (141.4–170.4)	88.9 ± 35.7 (80.8–98)	<0.001
Total cholesterol	186.4 ± 36 (178.4–194.4)	163.1 ± 35.9 (155.1–171.1)	<0.001
HDL	43 ± 10.6 (40.7–45.4)	54.9 ± 12.5 (52.1–57.7)	<0.001
LDL	119.8 ± 35.3 (112.2–128.2)	98.6 ± 33.3 (91–105.8)	<0.001
Creatinine	0.7 ± 0.2 (0.7–0.8)	0.7 ± 0.02 (0.7–0.7)	0.301
Uric acid	6.3 ± 6.9 (4.7–7.9)	4.5 ± 1.2 (4.2–4.8)	0.031
TSH	2.6 ± 1.3 (2.3–2.9)	2.8 ± 1.5 (2.4–3.1)	0.318
Insulinemia	24.6 ± 18.9 (20.3–29)	6.9 ± 3.05 (6.2–7.8)	<0.001

Abbreviations: BMI, body mass index; HDL, high‐density lipoproteins; LDL, low‐density lipoproteins; TSH, thyroid‐stimulating hormone.

Regarding MetS, its pre‐procedure prevalence was 66.2%, with a statistically significant decrease (*p* < 0.05) of 94.4%. This means that the prevalence of MetS 1 year after the procedure was only 3.7%. There was a greater number of patients with MetS in the LRYGB type procedure. In the same way, a discrimination by gender was made, which showed that there is a higher prevalence in the female sex (41.2%) and that this value is reduced to only 2.5% after the procedure (Table 3).

**Table 3:** Prevalence of metabolic syndrome before and after surgery, according to gender.

	Pre‐surgical			Follow‐up at 12 months		
	SGIT	LRYGB	Total	SGIT	LRYGB	Total
	*n* = 32 (%)	*n* = 48 (%)	*n* = 80 (%)	*n* = 32 (%)	*n* = 48 (%)	*n* = 80 (%)
Female	11 (34)	22 (45.8)	33 (41.2)	1 (3.1)	1(2)	2 (2.5)
Male	9 (28.1)	11 (22.9)	20 (25)	‐	1(2)	1 (1.2)

Abbreviations: LRYGB, Laparoscopic Roux–in–Y Gastric Bypass; SGIT, sleeve gastrectomy.

Additionally, and due to the importance of insulin resistance as a risk factor to generate MetS, we calculated it by means of the Homeostatic Model Assessment for Insulin Resistance (HOMA) score, showing that 77.5% of the total participants had this comorbidity and after the procedure it only persisted in 22.5%.

## 4 DISCUSSION

Obesity is a major public health problem in Colombia, which has been increasing rapidly in recent decades, and according to the latest figures from the National Survey of the Nutritional Situation (ENSIN) for 2015, there was a prevalence of obesity of 18.7% (about 10 million Colombians).^4^, ^13^ It has been associated with reduced life expectancy in young adults and is commonly associated with MetS,^14^ which leads to a significant increase in morbidity, mortality and cardiovascular risk. MetS is directly related to multiple pathologies, it is responsible for developing cardiovascular diseases in up to 25%, it increases the risk of DM2 by up to five times. Its prevalence in patients with coronary syndrome is up to 51%. In addition, it increases the risk up to five times of fatal outcomes in patients with cardiac pathologies.^15^, ^16^, ^17^, ^18^

The timely diagnosis of MetS is a valid tool for the assessment of cardiovascular risk that allows preventive actions to be carried out with the aim of reducing the morbidity and mortality associated with MetS.^19^ It should be detected and diagnosed early, especially in patients whose comorbidities directly influence the development of this syndrome, such as obesity.^20^

This study found that the unadjusted prevalence of MetS in patients with BMI > 30 who underwent MBS was 66.2% before the procedure, significantly higher than the national average. However, 1 year after the procedure the prevalence of MetS decreased by 94.4%, these results are similar to those found by Batsis et al.,^8^ in a retrospective population study in which they found a 58% decrease in the prevalence of MetS after undergoing MBS. These results are similar to other studies that have compared the reduction of MetS after mbS reporting a reduction between 56% and 76%.^21^, ^22^

Although recommended first‐line treatments for Mets do not include surgical intervention, MBS is an effective treatment for MetS that has a strong association with cases of obesity where early management has failed, resulting in rapid weight loss, and control of MetS‐associated comorbidities.^23^ Recent evidence suggests that MBS decreases overall mortality from all causes, including myocardial infarction and cancer.^24^ Sheng et al.,^25^ in a systematic review and meta‐analysis found that MBS significantly increased diabetes remission (relative risk [RR] = 5.90; 95% CI 3.75–9.28), reduced microvascular events (RR = 0.37; 95% CI 0.30–0.46), macrovascular (RR = 0.52; 95% CI 0.44–0.61) and mortality (RR = 0.21; 95% CI 0.20–0.21) compared to nonsurgical treatment.^25^

The most performed MBS procedure in Colombia is the LRYGB, followed by the SGIT. LRYGB is a mixed procedure, since it has the restrictive and poorly absorbed component,^26^, ^27^ this procedure shows adequate levels of safety. The risk of dying from surgery is <0.2% and the risk of serious complications such as bleeding, infections and thromboembolism is less than 5%.^28^ SGIT is a purely restrictive procedure, and the risk of complications and death are similar to that of LRYGB.^29^ Currently, one of the recommended procedures for the patient with obesity and MetS in whom conservative treatment has failed is LRYGB, since when the anatomical structure of the gastrointestinal tract is modified, the capacity of the stomach decreases and prevents the passage of food through the duodenum and proximal jejunum, decreasing the percentage of absorption of lipids and carbohydrates, impacting on a rapid, effective, and long‐term loss of weight.^30^

Although SGIT is not the ideal surgery for MetS, in our study the pre‐procedure prevalence was 25% and 1 year after the procedure it decreased to 2.5%, showing that it also has an important effect in reducing MetS. Our data were consistent with the available literature; however, it is important to clarify that as it is a purely restrictive procedure, its effect is not as effective as mixed or malabsorptive procedures.^31^

The evidence reported around the positive effect of MBS and weight loss and decrease in MetS, is associated with the remission of insulin resistance, accompanied by the restrictive component; in obesity, before the high load of glucose concentrations, the beta‐pancreatic cells have to respond with an abnormal hypersecretion of insulin, and because this constant stimulation, the failure of the pancreatic beta cells is generated, decreasing the production of insulin. After the MBS, a decrease in the concentration of glycemia has been reported so there is a decrease in the hypersecretion of insulin. It has also been reported decrease in blood pressure figures, and lipid profile, all these changes together decrease the cardiovascular risk of patients undergoing MBS.^32^

In this study, 77.5% of participants had pre‐procedure IR, and 1 year after MBS, 22.5% of participants still had IR. These results are consistent with other studies, such as those found by Cazzo et al.,^29^ in a retrospective cohort, reported a strong relationship between IR indices with a resolution rate of 88.5% (*p* < 0.0001) of the MetS.^29^

In Colombia, there are no studies conducted on MBS and its impact on MetS, we consider that our study is the first to measure the prevalence of MetS in patients undergoing MBS. However, as limitations, we mention that due to the retrospective nature of this study, it was not possible to perform a detailed analysis in different scenarios over time, to identify circumstantial patterns as reported by other authors. On the other hand, we did not include data related to complications derived from surgery. Therefore, it is necessary to develop more studies in our context that aim to evaluate the impact of MBS on the comorbidities of patients who are candidates for these procedures, to assess in the long term the real impact of this intervention on the population, based on disease indicators and health determinants.

## 5 CONCLUSIONS

Metabolic and bariatric surgery is an effective treatment for weight reduction, with a high impact in reducing the prevalence of metabolic syndrome and insulin resistance in the short and medium term in the Colombian population. However, it is important to carry out multiple studies evaluating whether this impact on MetS and insulin resistance is maintained over time.

## AUTHOR CONTRIBUTIONS

**Gonzalo A. Domínguez Alvarado**: Conceptualization; formal analysis; investigation; resources; software; supervision. **María C. Otero Rosales**: Data curation; methodology; project administration; resources; visualization; writing – original draft. **Julián C. Cala Duran**: Investigation; methodology; validation; visualization; writing – original draft. **Sergio Serrano‐Gómez**: Formal analysis; investigation; methodology; supervision; writing – original draft; writing – review and editing. **Tania Y. Carrero Barragan**: Methodology; project administration; supervision; writing – original draft; writing – review and editing. **Paula N. Domínguez Alvarado**: Investigation; project administration; writing – original draft; writing – review and editing. **Martha L. Ramírez**: Conceptualization; formal analysis; project administration; software; writing – review and editing. **Kamila Serrano‐Mesa**: Investigation; methodology; visualization; writing – original draft; writing – review and editing. **Ivan D. Lozada‐Martinez**: Methodology; project administration; supervision; validation; visualization; writing – review and editing. **Alexis R. Narvaez‐Rojas**: Methodology; project administration; writing – original draft; writing – review and editing. **Luis E. López Gómez**: Methodology; project administration; validation; writing – original draft; writing – review and editing.

## CONFLICT OF INTEREST STATEMENT

The authors declare no conflict of interest.

## ETHICS STATEMENT

It is an observational project based on anonymized secondary data. This study was reviewed and approved by the Ethics Committee of the Universidad Autónoma de Bucaramanga (no. INV2‐FO‐01).

## TRANSPARENCY STATEMENT

The lead author Alexis Rafael Narvaez‐Rojas affirms that this manuscript is an honest, accurate, and transparent account of the study being reported; that no important aspects of the study have been omitted; and that any discrepancies from the study as planned (and, if relevant, registered) have been explained.

## Data Availability

The authors confirm that the data supporting the findings of this study are available within the article.
